# Global infectious disease surveillance: bridge a 30-metre gap between the International Civil Aviation Organization and the World Anti-Doping Agency

**DOI:** 10.7189/jogh.15.03010

**Published:** 2025-01-31

**Authors:** Jie Huang, Mehmet Güllüoğlu, Ole Döring, Haidong Wang, Jiaying Li, Yao Liu

**Affiliations:** 1 School of Public Health and Emergency Management, Southern University of Science and Technology, Shenzhen, China; 2 Institute for Global Health and Development, Peking University, Beijing, China; 3 Türkiye Green Crescent Association, Istanbul, Türkiye; 4 Institute for Technology Futures, Karlsruhe Institute of Technology, Karlsruhe, Germany; 5 School for Foreign Language Studies, Hunan Normal University, Changsha, Hunan, China; 6 The School of Civil Engineering, The University of Sydney, New South Wales, Australia; 7 Department of Flight Standards, Civil Aviation Administration of China, Beijing, China

## Abstract

The global aviation industry faced unprecedented challenges during the COVID-19 pandemic. As a result, the international civil aviation industry now has strong incentives to prevent and control future pandemics. It is almost unbelievable that the headquarters of the International Civil Aviation Organization (ICAO) is located right next to the headquarters of the World Anti-Doping Agency (WADA). While the vision and mission of these two organizations may seem completely different, we propose that ICAO could adopt a system similar to that of WADA to enhance its contribution to global infectious disease surveillance.

## CIVIL AVIATION DURING COVID-19

The global aviation industry faced unprecedented challenges during the COVID-19 pandemic. While visible losses, such as job cuts in aviation and declines in airport duty-free sales, were substantial, the invisible disruptions – such as impacts on international collaboration and family reunions – were even greater. Although some measures may have been overreactions, others were well justified, as numerous studies have shown that international flights are closely linked to the spread of airborne infectious diseases [1,2].

Therefore, the international civil aviation industry has strong incentives to prevent and control future pandemics, even for its own survival and sustainability. On par with scientific publication stressing the importance of airports and aircrafts in global infectious disease surveillance [3], technological advances, including wastewater sampling from airports and airplanes, are becoming a reality [4]. The global nature of air travel makes the aviation industry a key player in both the spread and surveillance of infectious diseases.

## INTERNATIONAL CIVIL AVIATION AND WORLD ANTI-DOPING

**Figure 1:**
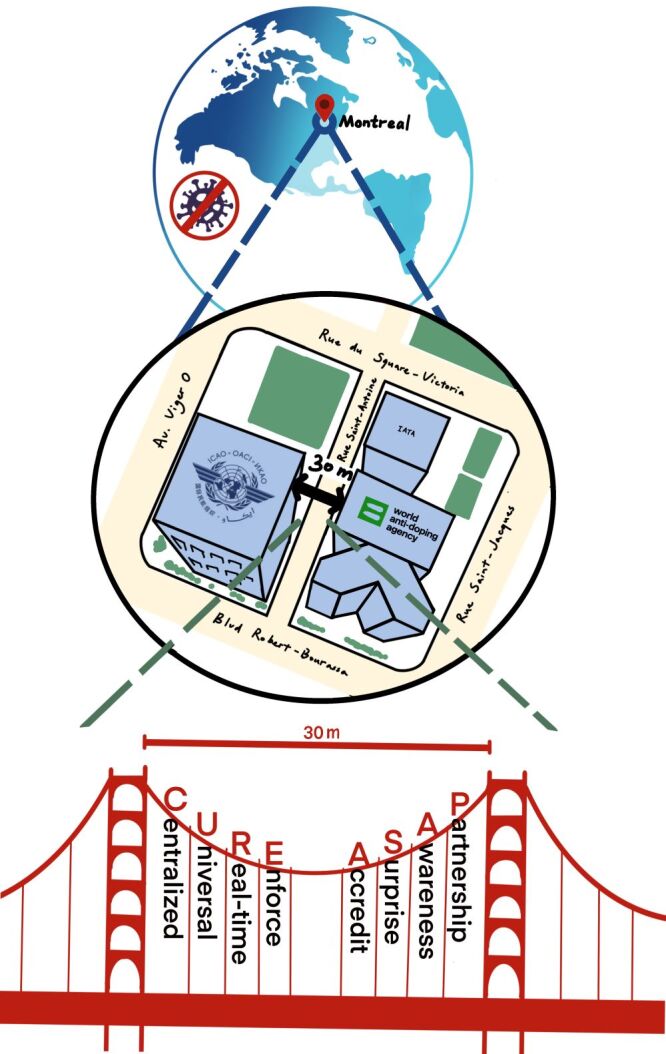
Bridge a 30-metre gap between International Civil Aviation Organization and World Anti-Doping Agency.

What more can be done to ensure that the international civil aviation industry is prepared to prevent and combat the next pandemic? With a virtual tour of the headquarters of the International Civil Aviation Organization (ICAO), we discovered that the answer may lie just next door – at the headquarters of the World Anti-Doping Agency (WADA). It is almost unbelievable that these two headquarters are located right next to each other, with the two nearest points only 30 m apart (**Figure 1**). This proximity feels like a divine will, and there are clear parallels between keeping drugs out of athletics and keeping infectious disease hotspots out of human society. After all, the ICAO had actively pursued the Public Health Corridor (PHC) strategy, a multilayer approach to mitigate the spread of COVID-19 through air travel.

According to a definition posted at the official website of the General Administration of Sport of China, out-of-competition doping testing is translated as ‘flying doping testing (飞行药检)’ [5]. The Chinese term literally reflects that such testing is carried out by and on personnel who frequently travel by air. It vividly highlights the close connection between doping control and civil aviation. To make global infectious disease surveillance work similarly to global anti-doping efforts, principles from WADA’s model should be adapted to the field of infectious disease detection, response, and prevention. Imagine if the international community had stored environmental and biological samples long before the COVID-19 pandemic began; the search for the origins of the SARS-CoV-2 virus would have been more scientific and objective.

## A ‘CURE-ASAP’ FRAMEWORK FOR ‘ICAO + WADA’

We propose a framework that could guide ICAO in adopting a system similar to WADA's. The first letters of the eight key points form the acronym ‘CURE-ASAP’. This framework should empower ICAO to play a leading role in global infectious disease surveillance.

### Centralised governance

The WADA provides a unified, globally recognised body that coordinates anti-doping activities, develops standards, and oversees compliance. A similar structure for infectious disease surveillance could be created through close collaboration between the World Health Organization (WHO) and ICAO.

### Universal protocol

The WADA applies universally recognised testing procedures across all sports, with specific tests for various substances. Likewise, a global infectious disease surveillance system would require universal diagnostic standards, applicable to potential hotspots such as airports worldwide.

### Real-time reporting

The WADA employs a global ADAMS database where testing results are stored and accessed by relevant authorities [6]. Real-time data sharing has already been integrated into the civil aviation industry, with services like FlightRadar24 (www.flightradar24.com) allowing millions to track airplanes. Through collaboration between ICAO and WHO, it would be feasible for the aviation industry to report infectious disease risks in real-time.

### Enforcement mechanism

The WADA enforces compliance through strict sanctions on athletes and sports organisations that violate anti-doping rules. In a global infectious disease surveillance system, enforcement could involve international pressure, economic penalties, or restricted access to global funding for countries that fail to comply with testing and reporting protocols.

### Accredited laboratories

The WADA has a network of accredited laboratories that perform standardised testing. For infectious disease surveillance, countries could develop a network of WHO-accredited laboratories, adhering to standardised protocols for testing and data reporting. With ICAO’s support, such laboratories could be established at major airports, with portable versions that could be used on long-haul international flights.

### Surprise testing

The WADA uses surprise testing as a key tactic in anti-doping efforts. Similarly, disease surveillance could include random audits or screenings at critical locations, such as major airports and sewage processing plants. Just as WADA has the authority to demand a doping test from any athlete, at any time, and anywhere, a global infectious disease surveillance system would need similarly empowered personnel with the authority to inspect and cross-check.

### Awareness campaigns

The WADA’s anti-doping programmes include robust education campaigns to help athletes avoid inadvertent violations. Similarly, global disease surveillance could be paired with public health campaigns to educate the public and policymakers about the importance of early reporting and symptom recognition.

### Partnership and pride

A framework of this scale would require close partnerships among stakeholders, including international and national public health agencies, non-governmental organisations (NGOs), and the private sector, to ensure sustainability. One key incentive for partnership would be national pride: a doping scandal can tarnish a nation’s reputation, motivating governments to prioritise compliance.

### Key steps for implementation

The following three steps are key to the successful implementation and long-term survival of the proposed framework.

#### Laws and legitimacy

The experiences and lessons learned by the international civil aviation sector from the COVID-19 pandemic must inform future strategies. Strengthening international health laws, such as the International Health Regulations (IHR), with enforcement mechanisms similar to those used in anti-doping violations could enhance global health security.[7]

#### Technological developments and deployments

Information sharing systems such as the GISAID database need to made more user-friendly [8]. Artificial intelligence (AI) should be more widely adopted to build tools such as Blue Dot, which combines aviation data and other data sources to issue early warning about potential epidemic/pandemic threats [9]. A joint WHO-ICAO panel could oversee the development of a network of accredited laboratories, similar to the WADA accredited laboratories. With ICAO’s support, such laboratories could be situated at major airports, and portable versions could even be installed and operated on long-distance international flights.

#### Ethical concerns

Adapting WADA’s principles to infectious disease surveillance could facilitate a reliable and rapid global detection system for health threats. However, its success would depend on overcoming challenges related to national sovereignty, data sharing, and equitable access to resources. Fortunately, proposals and progress have already been made toward a ‘pandemic treaty’ focusing on data sharing and transparency [10]. COVID-19 was eventually controlled with vaccines, in no small part because virus samples and sequencing data were shared quickly and continuously

## SPECIFIC CALLS TO STAKEHOLDERS

Policymakers at global, national, and regional levels should support the integration of aviation industry infrastructure into global health surveillance systems by strengthening legal frameworks for international health regulations and supporting funding for technological advancements in disease detection.

### The ICAO

The ICAO must take a leading role in creating global infrastructure for infectious disease surveillance. By collaborating with the WHO, ICAO can play a pivotal part in developing universal protocols and real-time reporting systems to monitor and prevent the spread of infectious diseases across borders.

### The WHO

The WHO should work closely with ICAO to establish standardised diagnostic testing and real-time disease reporting systems, leveraging the aviation network for rapid response. Additionally, WHO should advocate for the integration of disease surveillance mechanisms into the IIHR to ensure comprehensive global health security.

### The WADA

The WADA should collaborate with ICAO and WHO to adapt its framework to the context of infectious disease detection and response, providing guidance on the establishment of accredited laboratories and real-time data sharing. WADA’s model of centralised governance, universal protocols, and surprise testing can serve as a blueprint for global infectious disease surveillance.

### NGOs

NGOs, particularly those focused on global health, should advocate for policy changes and serve as intermediaries to ensure equitable access to disease surveillance tools across nations.

### Private sectors

Private sectors involved in aviation technology, data-sharing platforms, and health care should be engaged as strategic partners in building the necessary infrastructure for global infectious disease surveillance.
